# Supplementary material for: A Combined Experimental and Theoretical Study of Nitrofuran Antibiotics: Crystal Structures, DFT Computations, Sublimation and Solution Thermodynamics
Source: Molecules. 2021 Jun 5;26(11):3444. doi: 10.3390/molecules26113444 (PMC8201255; doi:10.3390/molecules26113444)
Supplement: Supplementary file 1 [file molecules-26-03444-s001.zip › molecules-1214432-supplementary.pdf]

Supplementary material

for

A combined experimental and theoretical study of nitrofuran antibiotics: crystal structures, DFT computations, sublimation and solution thermodynamics

*Manin A.N.,[1] Drozd K.V.,[1] Voronin A.P.,[1] Churakov A.V.,[2] Perlovich G.L.[1]**

[1]G.A. Krestov Institute of Solution Chemistry of the Russian Academy of Sciences, 1 Akademicheskaya St., Ivanovo, 153045, Russian Federation

[2]Institute of General and Inorganic Chemistry, Russian Academy of Sciences, 31 Leninsky Prosp., Moscow, 119991, Russian Federation

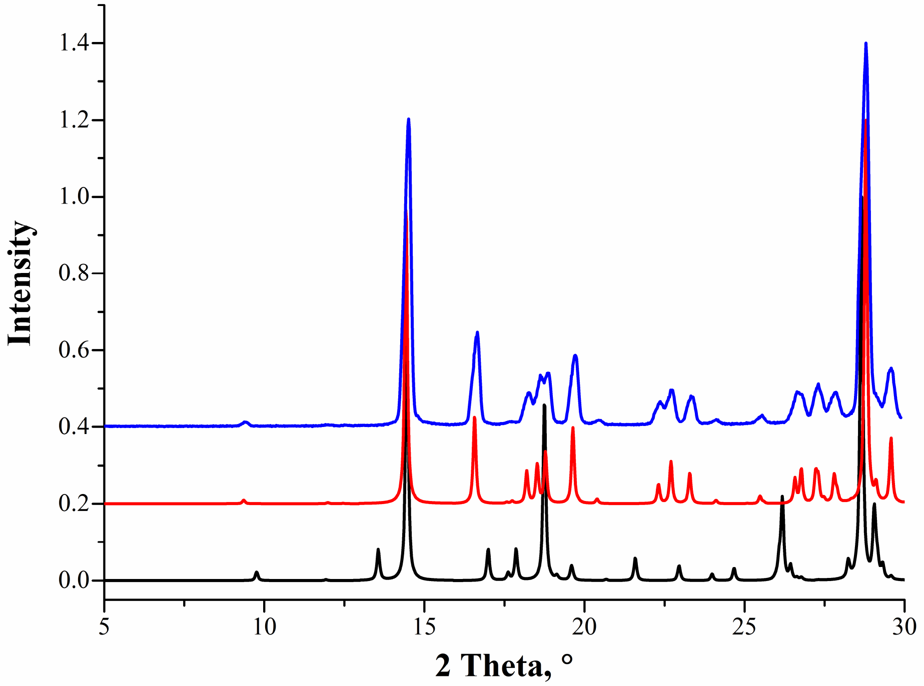

Fig. S1. An overlay of powder X-ray diffractograms of NFT: calculated based on the single-crystal data (triclinic α-polymorph (black), monoclinic β-polymorph (red)) and experimental diffractogram (blue).

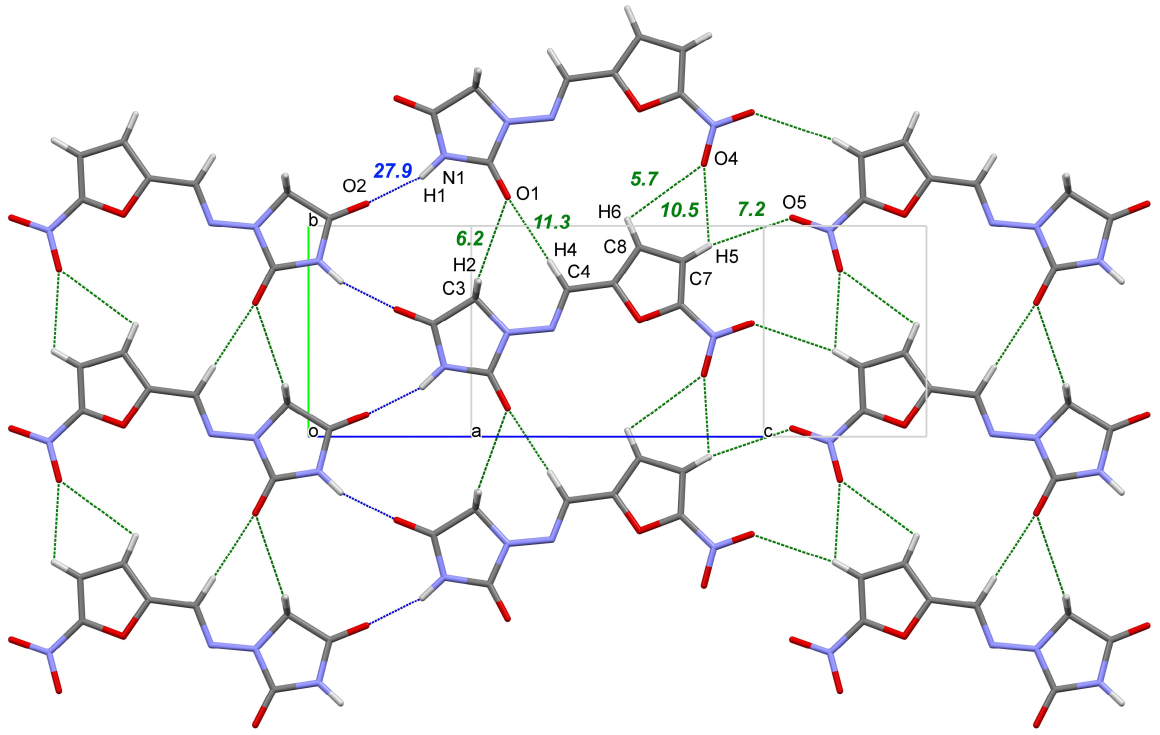

(a)

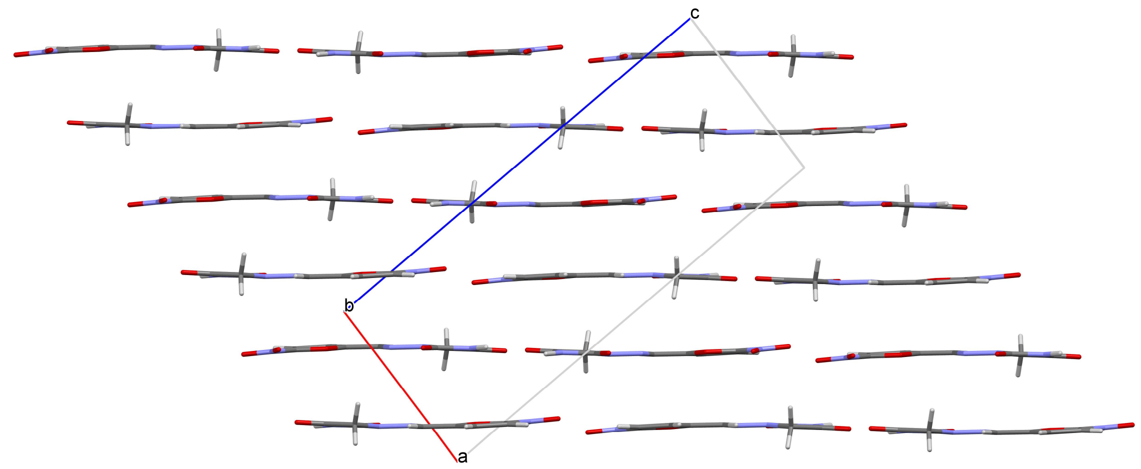

(b)

Fig. S2. Crystal structure of NFT (β-polymorph): (a) a 2D layer of NFT molecules formed by N−H···O (blue dots) and C−H···O bonds (green dots); (b) packing of 2D layers in the crystal. The numbers indicate the energy of H-bonds estimated using eq. (6) in kJ·mol^{-1}.

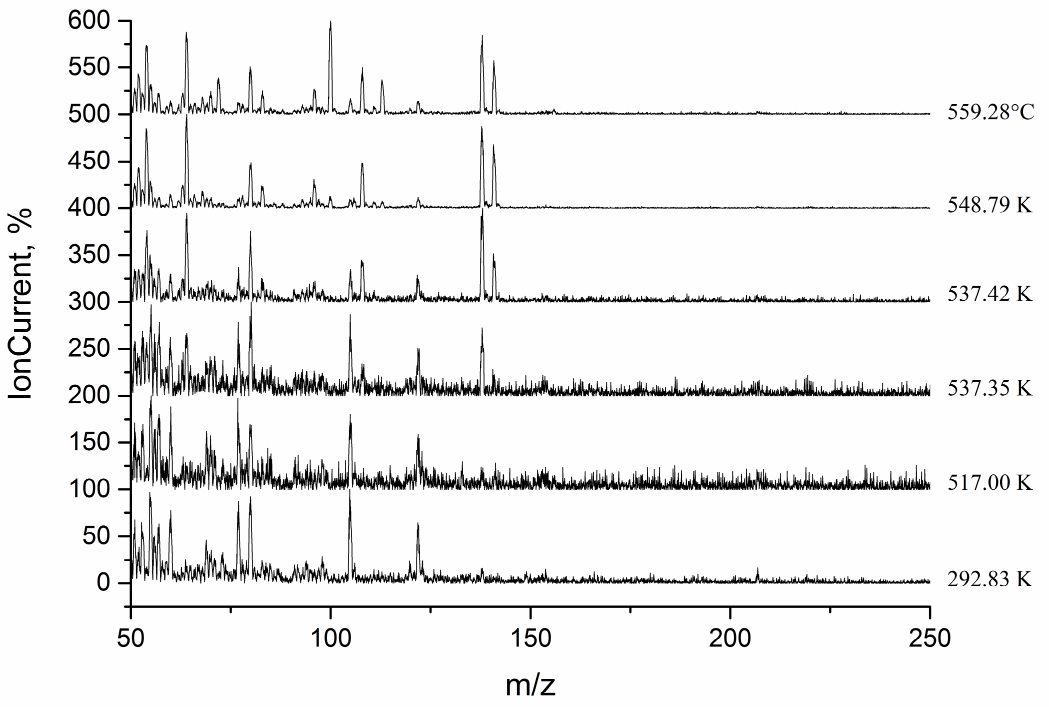

(a)

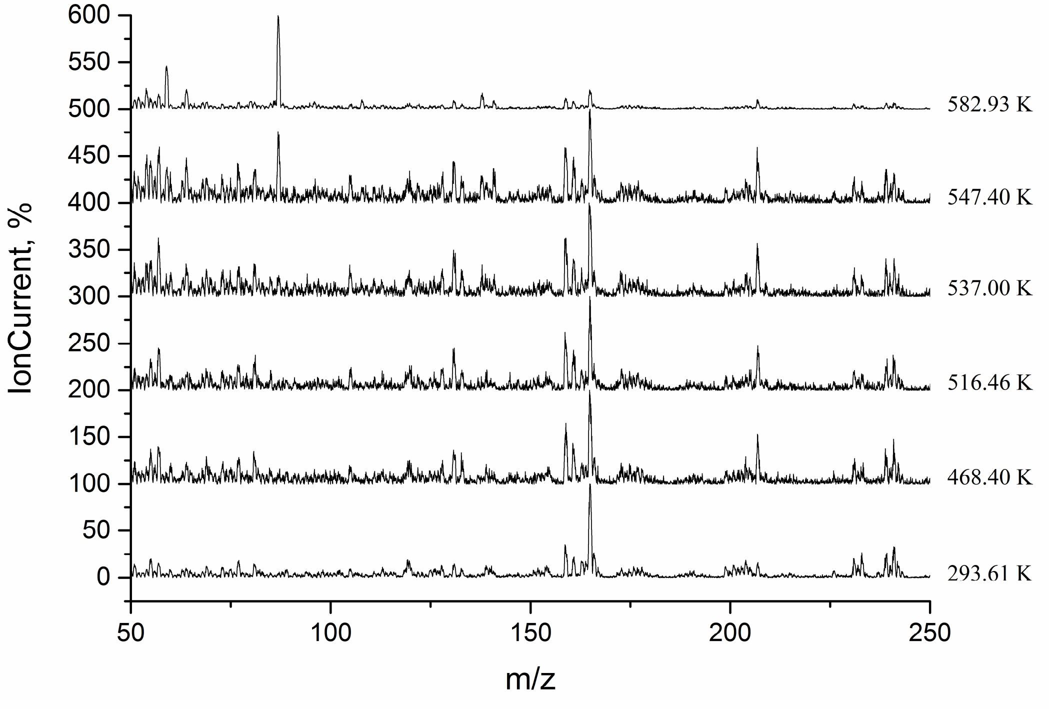

(b)

Fig. S3. Mass-spectra of (a)NFT and (b) FZL.

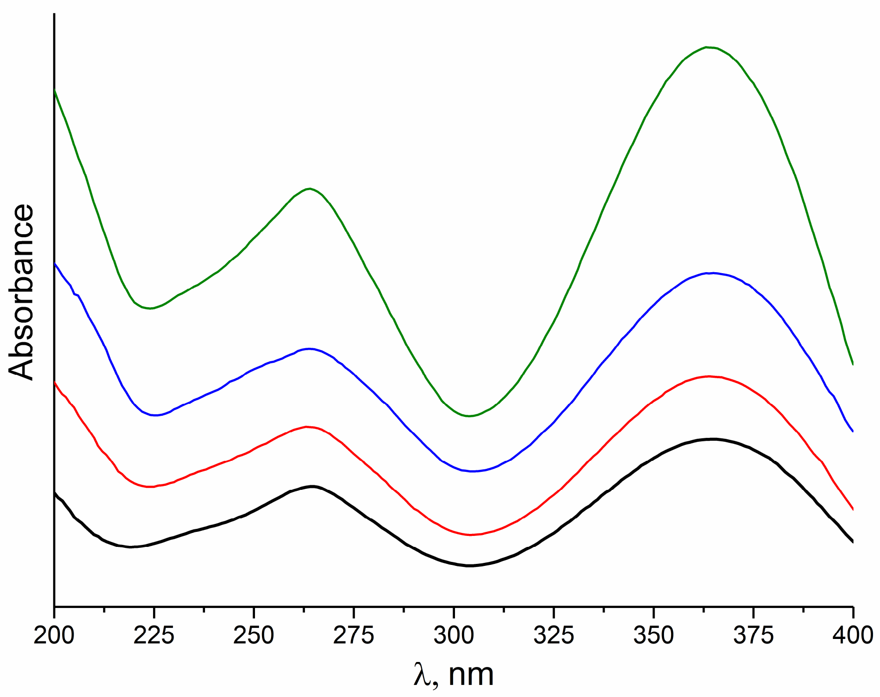

(a)

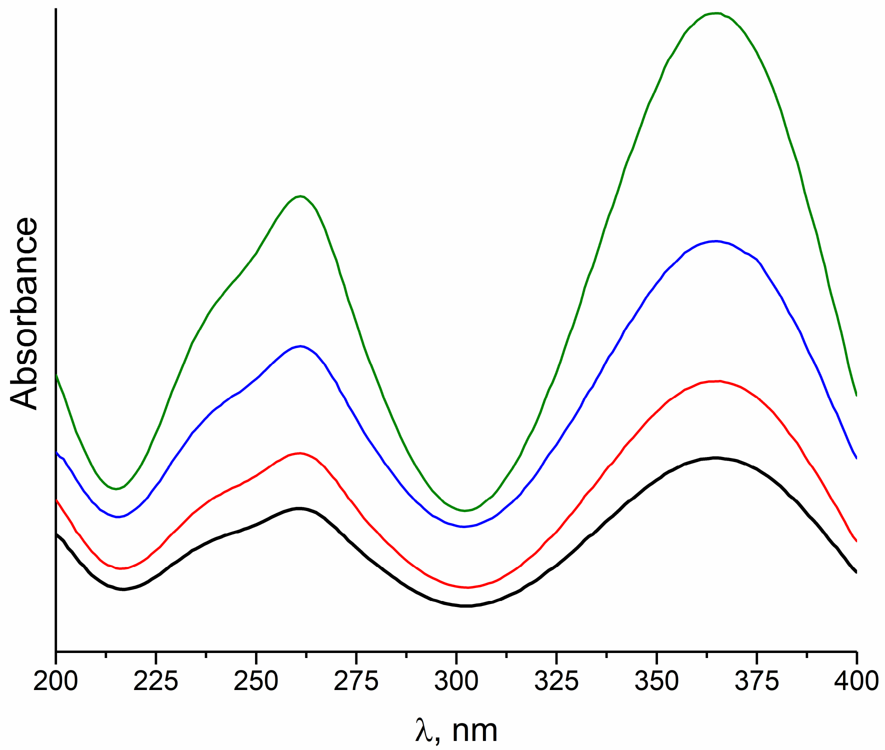

(b)

Fig. S4. UV absorption spectra before (black) and after the sublimation experiments of (a) NFT (red – at 438.6 K, blue – at 453.7 K, green – at 466.3 K) and (b) FZL (red – at 426.6 K, blue – at 437.9 K, green – at 443.4 K).

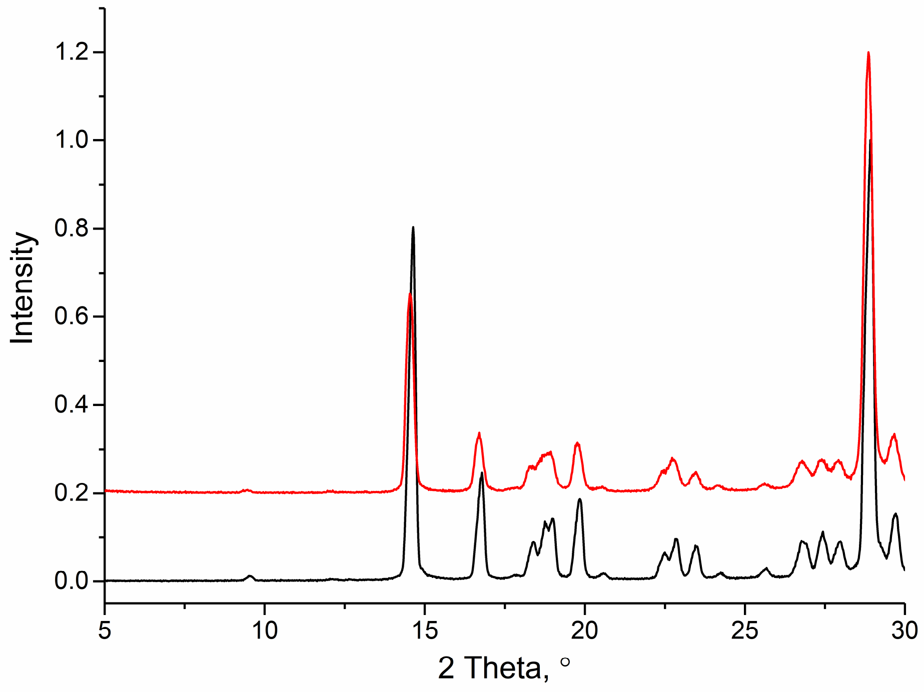

(a)

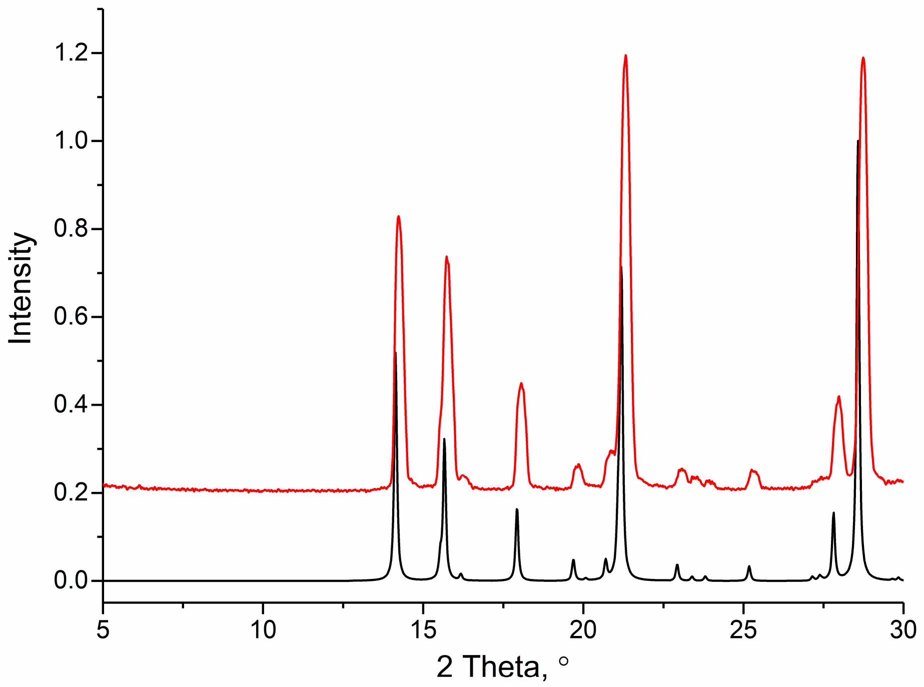

(b)

Fig. S5. An overlay of powder X-ray diffractograms of (a) NFT and (b) FZL before (black) and after (red) the sublimation experiments.

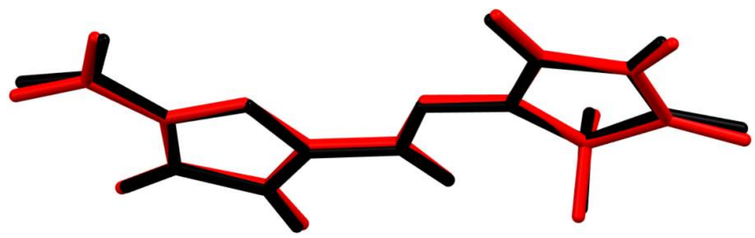

(a)

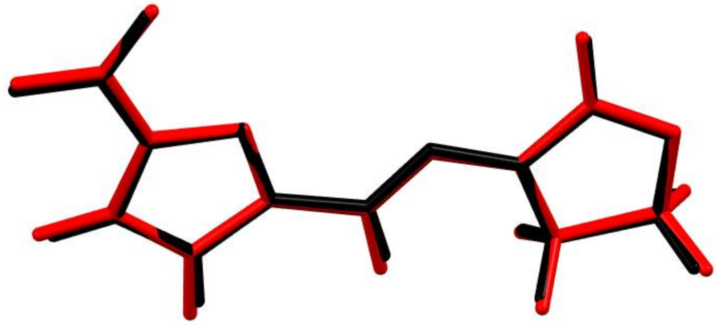

(b)

Fig. S6. An overlay of the single crystal (black) and optimized (red) structures of (a) NFT and (b) FZL.

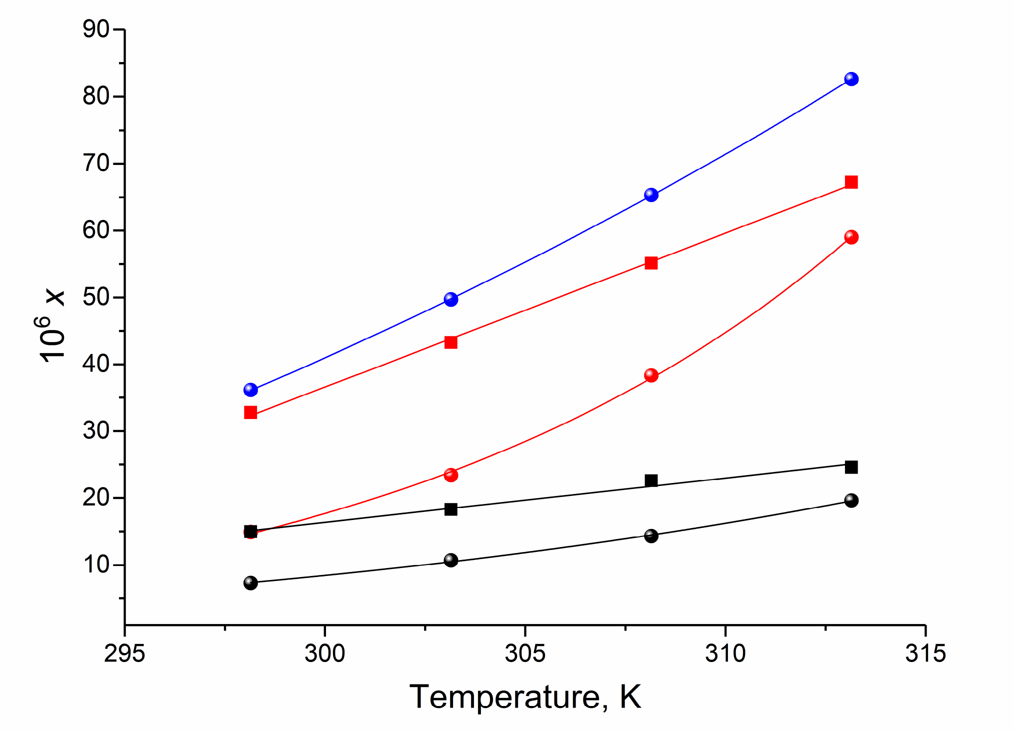

(a)

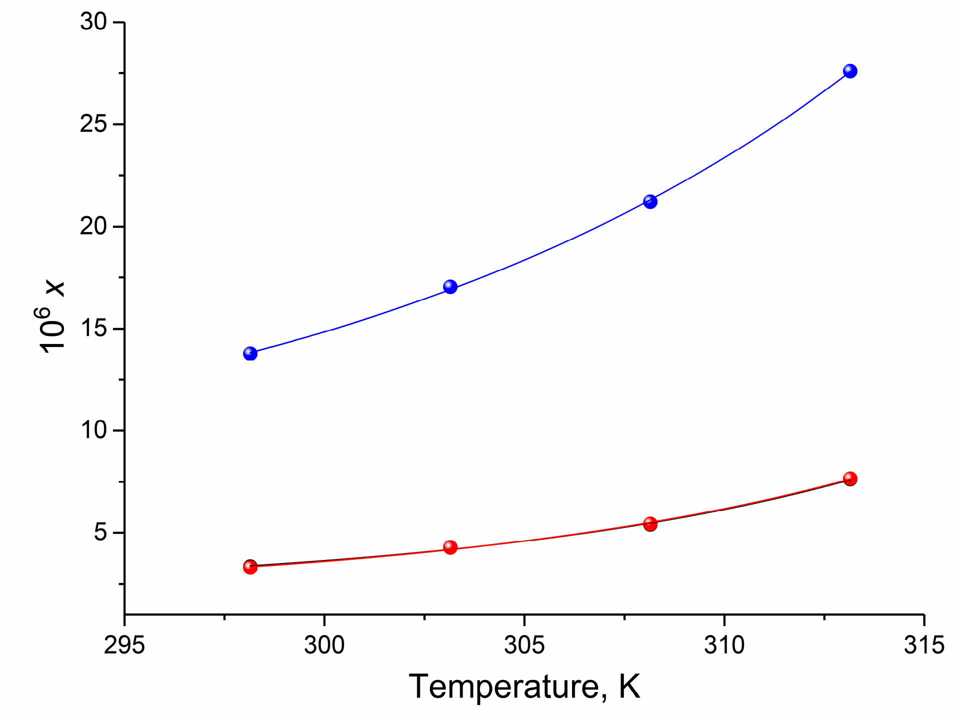

(b)

Fig. S7. Mole fraction solubility (X_2) of (a) NFT (●- in buffer pH 2.0 (NFT monohydrate), ■- in buffer pH 2.0 (NFT anhydrate), ●- in buffer pH 7.4 (NFT monohydrate), ■- in buffer pH 7.4 (NFT anhydrate), ●- in 1-octanol) and (b) FLZ (●- in buffer pH 2.0, ●- in buffer pH 7.4, ●- in 1-octanol) at different temperatures.

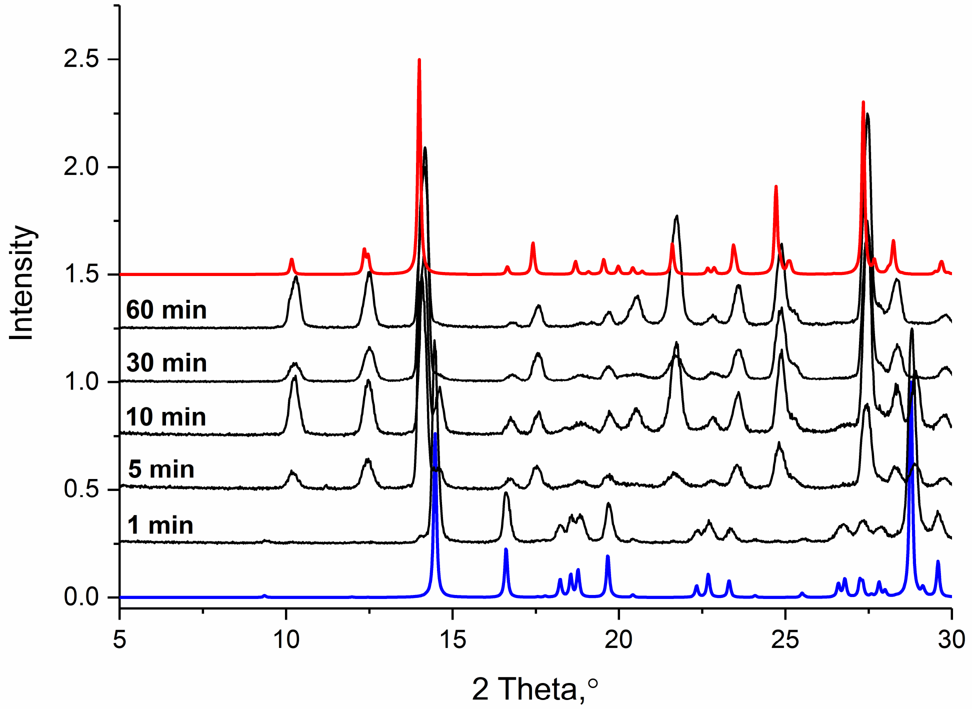

Fig. S8. PXRD analysis of the residual materials after solubility experiment of NFT in buffer pH 7.4 at 298.15 K from 1 to 60 minutes of experiment (PXRD of NFT β-polymorph (blue), NFT monohydrate *Pbca* form calculated based on the single-crystal data(red)).

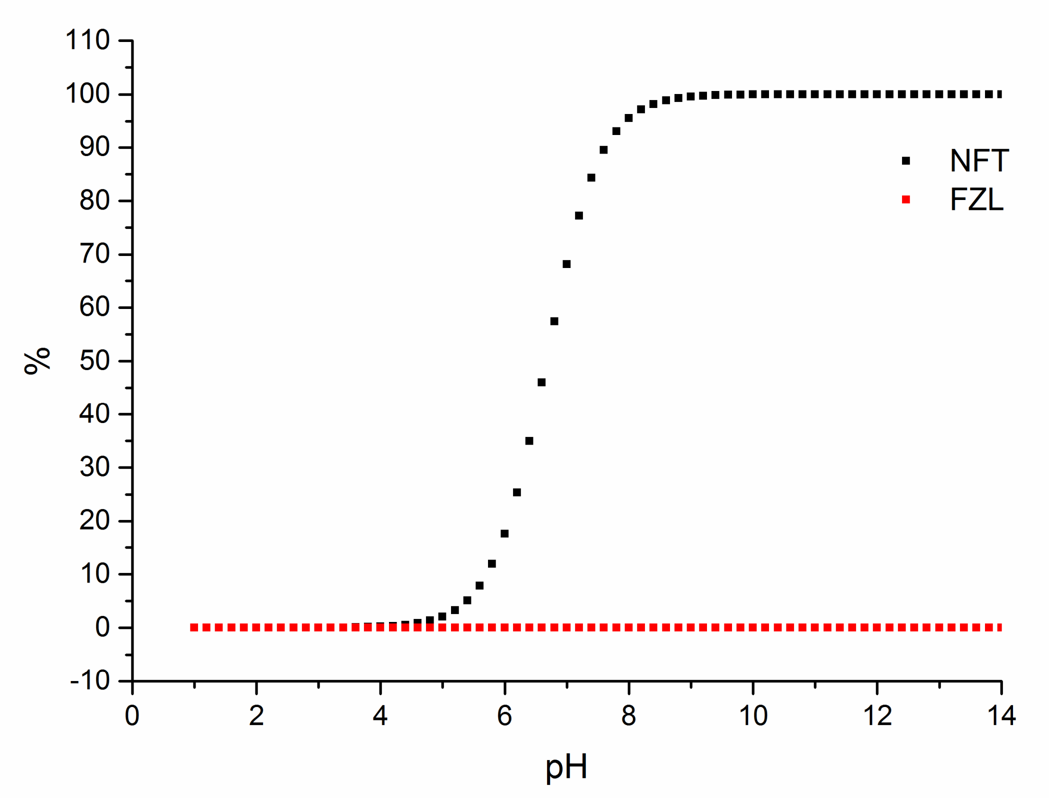

Fig. S9. Distribution diagram of the different species of NFT and FZL as a function of pH at 298.15 K (pK_a(NFT) 6.67 [1]; pK_a(FZL) -2.4 (calc. in ChemAxon))

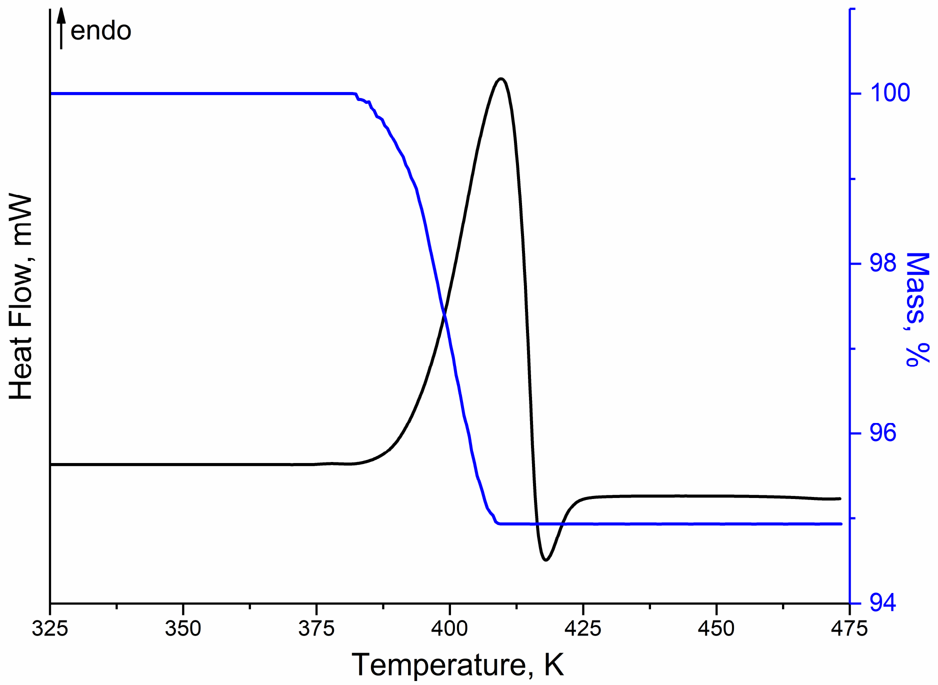

Fig. S10. DCS/TG curves of NFT monohydrate obtained after solubility experiment in buffer pH 7.4 at 298.15 K.

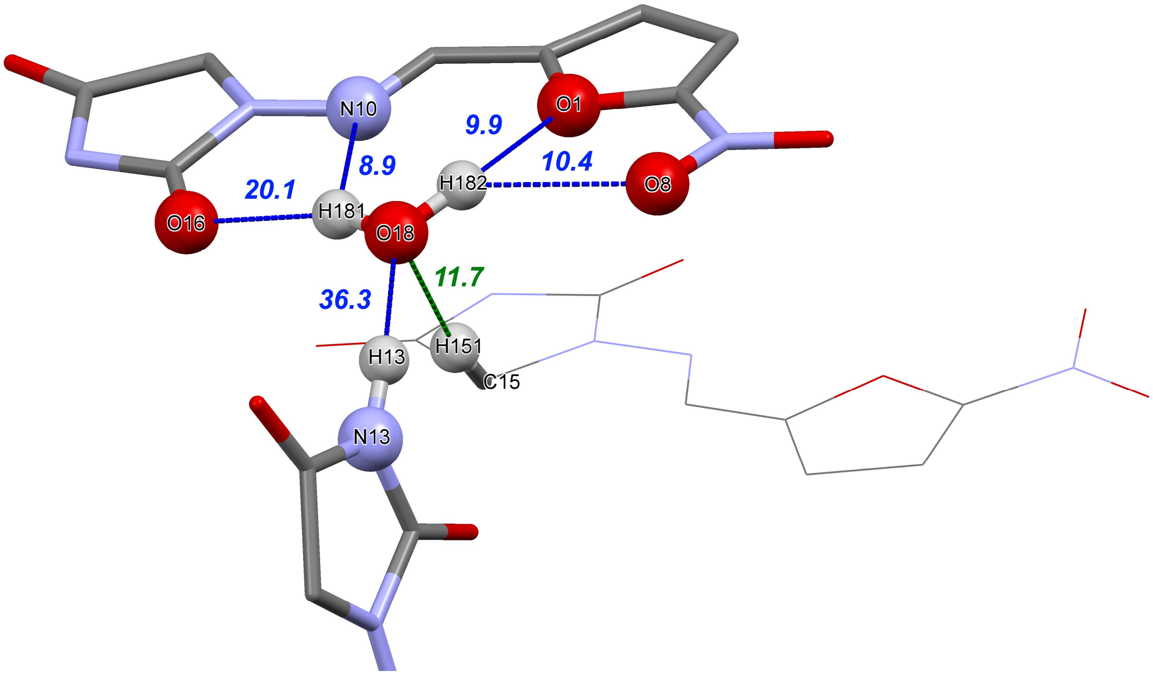

Fig. S11. Conventional (blue dotted lines) and non-conventional (green dotted line) hydrogen bonds formed by water molecules in the [NFT+H_2O] (1:1) crystal. The numbers denote the interaction energies estimated from QTAIMC analysis using Eq. (6). For clarity, only a part of the bottom NFT molecule is shown and the molecule in the back is displayed in a wireframe style.

Table S1. Density of the investigated solvents at different temperatures and constant pressure (p = 0.1 MPa)[a]

Solvent	ρ/g·cm^{-3}				
	293.15 K	298.15 K	303.15 K	308.15 K	313.15 K
Buffer pH 2.0[b]	1.0035	1.0023	1.0008	0.9992	0.9973
Buffer pH 7.4[c]	1.0060	1.0048	1.0033	1.0016	0.9998
1-Octanol	0.8251	0.8217	0.8183	0.8148	0.8114

[a]Density data for all solvents were taken from Ref. [2]

[b]Composition of aqueous buffer pH 2.0: KCl (6.57 g in 1 L) and 0.1 mol·L^{-1} hydrochloric acid (119.0 mL in 1 L);

[c]Composition of aqueous buffer pH 7.4: KH_2PO_4 (9.1 g in 1 L) and $Na_2HPO_4 \cdot 12H_2O$ (23.6 g in 1 L).

Standard uncertainties for mass salt (m) and volume of solution (V): $u(m)$ = 5 mg, u(V) = 0.5 mL.

Standard uncertainities: $u(T)$ = 0.15 K, $u(p)$ = 3 kPa and $u(\rho)$= 0.002 g·cm^{-3}.

Table S2. Transpiration experiment parameters, vapor pressure of the investigated compounds at different temperatures

T, K	m, mg	$V(N_2)$, dm^3	T_a, K	Flow, $dm^3 \cdot h^{-1}$	p, Pa	ln(p)
Nitrofurantoin (crystal)						
ln(p) = (33.6± 0.2) – (16832 ± 80) / T; σ = $1.07 \cdot 10^{-2}$; r = 0.99946; F = 44109; n = 25						
430.6	$3.22 \cdot 10^{-3}$	7.86	297.5	1.62	$4.25 \cdot 10^{-3}$	-5.46
432.4	$3.86 \cdot 10^{-3}$	7.86	297.7	1.62	$5.10 \cdot 10^{-3}$	-5.28
434.5	$4.80 \cdot 10^{-3}$	8.18	297.8	1.62	$6.10 \cdot 10^{-3}$	-5.10
436.3	$5.17 \cdot 10^{-3}$	7.89	297.5	1.62	$6.80 \cdot 10^{-3}$	-4.99
438.6	$6.63 \cdot 10^{-3}$	8.18	297.7	1.62	$8.42 \cdot 10^{-3}$	-4.78
440.6	$7.24 \cdot 10^{-3}$	7.32	297.5	1.62	$1.03 \cdot 10^{-2}$	-4.58
442.6	$8.07 \cdot 10^{-3}$	6.82	297.7	1.62	$1.23 \cdot 10^{-2}$	-4.40
444.3	$7.68 \cdot 10^{-3}$	5.78	297.6	1.62	$1.38 \cdot 10^{-2}$	-4.28
445.5	$1.57 \cdot 10^{-2}$	10.83	293.5	1.62	$1.48 \cdot 10^{-2}$	-4.21
446.8	$1.60 \cdot 10^{-2}$	9.78	293.5	1.62	$1.68 \cdot 10^{-2}$	-4.09
448.9	$1.90 \cdot 10^{-2}$	9.50	292.6	1.62	$2.04 \cdot 10^{-2}$	-3.89
451.1	$1.93 \cdot 10^{-2}$	7.83	298.0	1.62	$2.56 \cdot 10^{-2}$	-3.66
451.9	$2.27 \cdot 10^{-2}$	8.78	292.1	1.62	$2.64 \cdot 10^{-2}$	-3.63
453.7	$2.61 \cdot 10^{-2}$	8.75	293.7	1.62	$3.06 \cdot 10^{-2}$	-3.49
454.2	$2.45 \cdot 10^{-2}$	7.83	297.2	1.62	$3.24 \cdot 10^{-2}$	-3.43
455.4	$1.89 \cdot 10^{-2}$	5.48	295.9	1.62	$3.57 \cdot 10^{-2}$	-3.33
456.9	$2.40 \cdot 10^{-2}$	6.24	297.6	1.62	$4.00 \cdot 10^{-2}$	-3.22
457.6	$3.51 \cdot 10^{-2}$	8.59	295.3	1.62	$4.22 \cdot 10^{-2}$	-3.17
458.7	$3.75 \cdot 10^{-2}$	8.02	295.2	1.62	$4.81 \cdot 10^{-2}$	-3.03
460.7	$4.05 \cdot 10^{-2}$	7.51	293.0	1.62	$5.51 \cdot 10^{-2}$	-2.90
462.1	$3.71 \cdot 10^{-2}$	6.29	293.2	1.62	$6.03 \cdot 10^{-2}$	-2.81
463.7	$2.50 \cdot 10^{-2}$	3.75	292.9	1.62	$6.82 \cdot 10^{-2}$	-2.68
465.4	$3.40 \cdot 10^{-2}$	4.56	293.7	1.62	$7.63 \cdot 10^{-2}$	-2.57
466.3	$3.83 \cdot 10^{-2}$	4.67	293.0	1.62	$8.39 \cdot 10^{-2}$	-2.48
467.4	$3.89 \cdot 10^{-2}$	4.35	294.4	1.62	$9.20 \cdot 10^{-2}$	-2.39
Furazolidone (crystal)						
ln(p) = (30.9 ± 0.3) – (14671 ± 127) / T; σ = $3.90 \cdot 10^{-3}$; r = 0.99951; F = 13329; n = 15						
423.1	$1.71 \cdot 10^{-2}$	8.07	297.8	1.62	$2.33 \cdot 10^{-2}$	-3.76
424.6	$1.90 \cdot 10^{-2}$	7.71	297.5	1.62	$2.70 \cdot 10^{-2}$	-3.61
426.6	$2.11 \cdot 10^{-2}$	7.24	298.3	1.62	$3.21 \cdot 10^{-2}$	-3.44
428.6	$2.51 \cdot 10^{-2}$	7.40	298.3	1.62	$3.73 \cdot 10^{-2}$	-3.29
429.9	$1.44 \cdot 10^{-2}$	3.73	298.2	1.62	$4.25 \cdot 10^{-2}$	-3.16
431.3	$1.41 \cdot 10^{-2}$	3.32	299.0	1.62	$4.69 \cdot 10^{-2}$	-3.06
433.0	$1.78 \cdot 10^{-2}$	3.77	297.3	1.62	$5.17 \cdot 10^{-2}$	-2.96
435.1	$1.95 \cdot 10^{-2}$	3.35	297.4	1.62	$6.39 \cdot 10^{-2}$	-2.75
436.1	$2.35 \cdot 10^{-2}$	3.77	297.5	1.62	$6.85 \cdot 10^{-2}$	-2.68
437.9	$2.37 \cdot 10^{-2}$	3.43	297.8	1.62	$7.57 \cdot 10^{-2}$	-2.58
439.1	$2.63 \cdot 10^{-2}$	3.48	297.8	1.62	$8.29 \cdot 10^{-2}$	-2.49
440.7	$2.90 \cdot 10^{-2}$	3.32	298.0	1.62	$9.59 \cdot 10^{-2}$	-2.34
442.1	$3.39 \cdot 10^{-2}$	3.52	297.6	1.62	$1.06 \cdot 10^{-1}$	-2.25
443.4	$3.06 \cdot 10^{-2}$	2.88	298.1	1.62	$1.17 \cdot 10^{-1}$	-2.15
444.0	$3.77 \cdot 10^{-2}$	3.40	297.6	1.62	$1.22 \cdot 10^{-1}$	-2.11

Standard uncertainty for temperature $u(T)$ = 0.15 K
Relative standard uncertainty for pressure $u_r(p)$ = 0.05

Table S3. Comparison of calculated (B3LYP-D3(BJ,ABC)/6-31G(d,p) level of theory) and experimental thermodynamic properties of sublimation processes of NFT and FZL

Compound	**NFT**		**FZL**	
	Calc.	*Exp.*[a]	*Calc.*	*Exp.*[a]
[b]E_{latt} (0 K), kJ·mol^{-1}	139.6		121.9	
$C_p^{cr}(298.15K)$, J·mol^{-1}·K^{-1}	236.5	287.8[c]	223.7	286.4[c]
$C_p^{g}(298.15K)$, J·mol^{-1}·K^{-1}	223.3	243.9[d]	202.0	242.7[d]
$\int_0^{298.15}(C_p^g - C_p^{cr})dT$, kJ·mol^{-1}	-1.4		-3.0	
E_{latt} (298.15K), kJ·mol^{-1}	138.2		118.9	
ΔpV, kJ·mol^{-1}	2.4		2.5	
$\Delta_{cr}^{g}H_m^0(298.15K)$ - E_{latt}(0 K), kJ·mol^{-1}	1.0		-0.5	
$\Delta_{cr}^{g}H_m^0(298.15K)$, kJ·mol^{-1}	140.7	146.5±0.7	121.4	127.9±1.1
[e]E_{latt} (298.15K) (2RT scheme), kJ·mol^{-1}	144.6		126.4	
S^{cr}(298.15K), J·mol^{-1}·K^{-1}	265.4		252.5	
S^{g}(298.15K), J·mol^{-1}·K^{-1}	515.4		477.5	
$\Delta_{cr}^{g}S_m^0(298.15K)$, J·mol^{-1}·K^{-1}	250.0	205.5±2.8	225.0	180.6±4.6
$298.15 \cdot \Delta_{cr}^{g}S_m^0(298.15K)$, kJ·mol^{-1}	74.5	61.3	67.1	53.9
$\Delta_{cr}^{g}G_m^0(298.15K)$, kJ·mol^{-1}	66.1	85.2	53.7	74.1

[a] Data taken from Table 4.

[b] E_{latt}(0 K) was calculated using equation (7) using the interaction energies obtained from QTAIMC data according to equation (6)

[c] $C_p(298.15K)$ was calculated according to Chickos' additive scheme [3]

[d] $C_p(298.15K)$ was calculated from the Chickos's correlation equation [4]

[e] Calculated according to equation (8)

Table S4. Compounds structurally similar to NFT and FZL, selected from the literature

N	Compound	CAS number	$\Delta_{cr}^{g} G_m^0(298.15)$ kJ·mol^{-1}	$\Delta_{cr}^{g} H_m^0(298.15)$ kJ·mol^{-1}	T_{fus}, K	Ref.
1	2-imadazolidinone	120-93-4	42.6	83.7 ± 0.8	397.35	[5]
2	Succinimide	123-56-8	36.7	83.6 ± 1.5	400.05	[6]
3	2,4-Imidazolidinedione	461-72-3	57.8	116.3 ± 1.5	495.75	[7]
4	2-Thiohydantoin	503-87-7	60.2	119.1 ± 0.5	506.05	[7]
5	5-(4-nitrophenyl)-furan-2-carbaldehyde	7147-77-5	65.4	135.9 ± 2.4	479.75	[8]

Table S5. Metric and electron-density features of hydrogen bonds formed by water molecules in the [NFT+H_2O] (1:1) crystal and in the hypothetical [FZL+H_2O] (1:1) crystal

Interaction	D···A, Å | H···A, Å	∠D–H···A, °	ρ_b, a.u.	$\nabla^2\rho_b$, a.u.	G_b, a.u.	E_{int}, kJ·mol^{-1}
			**[NFT+H_2O] (1:1)**			
N13−H13···O18	2.763 | 1.782	165.68	0.042	0.130	0.032	**36.3**
O18−H181···O16	2.961 | 2.015	157.45	0.024	0.070	0.018	**20.1**
C15−H151···O18	3.725 | 2.730	172.40	0.015	0.039	0.010	**11.7**
O18−H182···O8	3.172 | 2.392	134.54	0.012	0.038	0.009	**10.4**
O18−H182···O1	3.148 | 2.245	150.02	0.010	0.038	0.009	**9.9**
O18−H181···N10	3.211 | 2.499	126.07	0.010	0.036	0.008	**8.9**
Total energy						**97.2**
			**[FZL+H_2O] (1:1)**			
O18−H181···O16	2.859 | 1.966	151.15	0.024	0.073	0.018	**20.7**
C15−H151···O18	3.371 | 2.285	170.77	0.016	0.037	0.010	**11.3**
O18−H182···O1	3.125 | 2.336	138.12	0.010	0.039	0.009	**10.1**
O18−H181···N10	3.192 | 2.473	130.32	0.011	0.036	0.008	**9.0**
Total energy						**51.1**

References:

1. Domańska, U.; Pobudkowska, A.; Pelczarska, A.; Żukowski, Ł. Modelling, solubility and pKa of five sparingly soluble drugs. *Int. J. Pharm.* **2011**, *403*, 115–122, doi:https://doi.org/10.1016/j.ijpharm.2010.10.034.
2. Blokhina, S.; Sharapova, A.; Ol'khovich, M.; Perlovich, G. A thermodynamic study of sublimation, dissolution and distribution processes of anti-inflammatory drug Clonixin. *J. Chem. Thermodyn.* **2019**, *132*, 281–288, doi:https://doi.org/10.1016/j.jct.2019.01.006.
3. Chickos, J.S.; Acree, W.E. Enthalpies of Sublimation of Organic and Organometallic Compounds. 1910–2001. *J. Phys. Chem. Ref. Data* **2002**, *31*, 537–698, doi:10.1063/1.1475333.
4. Chickos, J.S.; Hosseini, S.; Hesse, D.G.; Liebman, J.F. Heat capacity corrections to a standard state: a comparison of new and some literature methods for organic liquids and solids. *Struct. Chem.* **1993**, *4*, 271–278, doi:10.1007/BF00673701.
5. Ribeiro da Silva, M. das D.M.C.; Ribeiro da Silva, M.A. V; Freitas, V.L.S.; Roux, M.V.; Jiménez, P.; Temprado, M.; Dávalos, J.Z.; Cabildo, P.; Claramunt, R.M.; Elguero, J. Structural studies of cyclic ureas: 1. Enthalpies of formation of imidazolidin-2-one and N,N′-trimethyleneurea. *J. Chem. Thermodyn.* **2008**, *40*, 386–393, doi:https://doi.org/10.1016/j.jct.2007.08.004.
6. Meng-Yan, Y.; Pilcher, G. Enthalpies of combustion of succinic anhydride, glutaric anhydride, and glutarimide. *J. Chem. Thermodyn.* **1990**, *22*, 893–898, doi:https://doi.org/10.1016/0021-9614(90)90177-R.
7. Silva, A.L.R.; Cimas, Á.; Vale, N.; Gomes, P.; Monte, M.J.S.; Ribeiro da Silva, M.D.M.C. Experimental and computational study of the energetics of hydantoin and 2-thiohydantoin. *J. Chem. Thermodyn.* **2013**, *58*, 158–165, doi:https://doi.org/10.1016/j.jct.2012.10.010.
8. Dibrivnyi, V.; Sobechko, I.; Puniak, M.; Horak, Y.; Obushak, M.; Van-Chin-Syan, Y.; Andriy, M.; Velychkivska, N. Thermodynamic properties of 5(nitrophenyl) furan-2-carbaldehyde isomers. *Chem. Cent. J.* **2015**, *9*, 67, doi:10.1186/s13065-015-0144-x.
